# Diagnostic Accuracy of Subtraction Coronary CT Angiography in Severely Calcified Segments: Comparison Between Readers With Different Levels of Experience

**DOI:** 10.3389/fcvm.2022.828751

**Published:** 2022-03-21

**Authors:** Fang Li, Qing He, Lixue Xu, Yan Zhou, Yufei Sun, Zhenchang Wang, Yinghao Xu, Zhenghan Yang, Yi He

**Affiliations:** ^1^Department of Radiology, Beijing Friendship Hospital, Capital Medical University, Beijing, China; ^2^Department of Radiology, Beijing Chest Hospital, Capital Medical University, Beijing, China; ^3^Canon Medical Systems (China) Co. Ltd., Beijing, China

**Keywords:** coronary computed tomographic angiography, coronary artery disease, subtraction, coronary artery calcification, blooming and beam-hardening artifacts

## Abstract

**Purpose:**

Subtraction coronary CT angiography (CCTA) may reduce blooming and beam-hardening artifacts. This study aimed to assess its value in improving the diagnostic accuracy of readers with different experience levels.

**Method:**

We prospectively enrolled patients with target segment who underwent CCTA and invasive coronary angiography (ICA). Target segment images were independently evaluated by three groups of radiologists with different experience levels with CCTA using ICA as the standard reference. Diagnostic accuracy was measured by the area under the curve (AUC), using ≥50% stenosis as the cut-off value.

**Results:**

In total, 134 target segments with severe calcification from 47 patients were analyzed. The mean specificity of conventional CCTA for each group ranged from 22.4 to 42.2%, which significantly improved with subtraction CCTA, ranging from 81.3 to 85.7% (all *p* < 0.001). The mean sensitivity of conventional CCTA for each group ranged from 83.3 to 88.0%. Following calcification subtraction, the mean sensitivity decreased for the novice (*p* < 0.001) and junior (*p* = 0.017) radiologists but was unchanged for the senior radiologists (*p* = 0.690). With subtraction CCTA, the mean AUCs of CCTA significantly increased: values ranged from 0.53, 0.54, and 0.61 to 0.70, 0.74, and 0.85 for the novice, junior, and senior groups (all *p* < 0.001).

**Conclusion:**

Subtraction CCTA could improve the diagnostic accuracy of radiologists at all experience levels of CCTA interpretation.

## Introduction

Coronary computed tomography angiography (CCTA) has become a cost-effective first line technique in the diagnosis of patients with chest pain (1, 2). The diagnostic performance of CCTA varies depending on the level of experience of the reading physician. It has been proven that a greater level of experience leads to improvements in the diagnostic accuracy of CCTA (3, 4).

In addition, inter-reader variability in CCTA interpretation also differs according to the readers' levels of experience. Even with the use of the Coronary Artery Disease Reporting and Data System (CAD-RADS), the standardized reporting system for coronary artery diseases, the inter-reader agreement of expert readers was stronger than that of early career readers (5). Severe coronary calcification is a major challenge for readers in interpreting CCTA. The “blooming” and beam-hardening artifacts of severely calcified plaques may cause difficulties for readers in estimating coronary luminal stenosis. The degree of coronary artery stenosis with severe calcification is often overestimated or underestimated, and in these segments, the diagnostic accuracy of CCTA subsequently decreases (6, 7). To address this problem, a subtraction CCTA method was proposed to allow elimination of the artifacts from calcified plaques (8). Several studies indicated that subtraction CCTA improved the diagnostic accuracy and increased the diagnostic confidence of readers when evaluating severely calcified segments (9–14). However, to our knowledge, whether the diagnostic accuracy and diagnostic confidence of subtraction CCTA varies among readers with different levels of experience remains unknown.

To address this issue, our research aimed to evaluate the value of subtracting CCTA in diagnosing severely calcified coronary artery stenosis by investigating the impact of this method on the diagnostic accuracy and diagnostic confidence of readers with different levels of experience.

## Materials and Methods

### Study Population

Patients with suspected coronary artery disease were prospectively selected to undergo subtraction CCTA at our institution from October 2019 to June 2020. The inclusion criteria were as follows: (1) age ≥ 50 years, (2) basal or drug control heart rate (HR) ≤ 65 bpm, (3) no contraindications for iodinated contrast material, (4) no history of cardiac surgery, (5) sinus rhythm, no history of arrhythmia or heart failure, and (6) willingness to provide written informed consent. The exclusion criteria were as follows: (1) no severe calcifications, (2) no invasive coronary angiography (ICA) or ICA performed more than 1 month after subtraction CCTA, and (3) severe motion artifacts. This study was approved by the ethics committee of our hospital, and all patients provided informed consent.

### Conventional CCTA Data Acquisition

The CT scanner used in this study was a 320-detector row CT scanner (Aquilion ONE Vision Edition, Canon Medical Systems) with 0.5 mm detector elements, a rotation time of 275 ms, and the Adaptive Iterative Dose Reduction 3D (AIDR 3D) algorithm. A two-breath hold acquisition technique was used (8). A cardioselective beta-blocker (metoprolol 25–150 mg) was administered orally 1 h before scanning for individuals with a heart rate > 65 bpm and no contraindications to the substance. Tube voltage and tube current were determined by an automated exposure control function (^SURE^ Exposure) with target image noise with a standard deviation (SD) of 22 Hounsfield units. Depending on the body mass index (BMI), 100 or 120 kV was used for both scans. The phase window was set at 70–80% of the R-R interval. Iodinated contrast medium with an iodine concentration of 370 mg I/ml (Iopamidol-370, Bayer Schering Pharma, Berlin, Germany) was injected at a rate of body weight (kg) × 0.07 mL/s in 10 s (fixed), followed by a 50-mL saline chaser bolus.

### Subtraction CCTA

**Figure 1:**
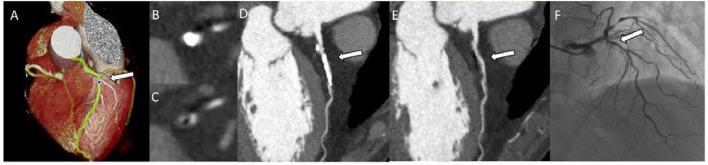
Patient showing stenosis regrading in conventional CCTA and subtraction. VR **(A)**, Axial **(B)**, and CPR **(D)** images of proximal left anterior descending artery with severe calcification on conventional CCTA. Axial **(C)** and CPR **(E)** images for subtraction CCTA. The ICA image **(F)**. The arrow indicates calcified plaque with or without subtraction CCTA, and the same lesion on ICA. CCTA, coronary computed tomography angiography; VR, Volume rendered; CPR, curved planar reconstruction; ICA, invasive coronary angiography.

Canon Sure Subtraction Coronary software was used for subtraction CCTA as previously reported (8). First, non-contrast and contrast images were reconstructed at 70–80% phases at 1% intervals to obtain the best-quality image sequence. Non-contrast and contrast image sequences without motion artifacts were selected as the best-quality image sequences for registration. The registration was performed in two steps. In the first step, a global non-rigid registration was performed. In the next step, a local rigid registration for the targeted coronary segment followed. Consequently, the subtraction images obtained. An example is shown in Figure 1.

### Image Analysis

Canon Workstation perfusion software (Vitrea fX, Version 40693, Vital Images, Minnetonka, MN and Canon Medical Systems, Otawara, Japan) was used to post-process the conventional and subtraction CCTA images. Eight radiologists, including three novices (who had performed between 50 and 100 CCTA interpretations), two junior radiologists (who had performed ~500 CCTA interpretations) and 3 senior radiologists (who had performed >1,000 CCTA interpretations), independently read the CCTA images twice. The first time, only conventional CCTA images were available; the second time, both conventional and subtraction CCTA images were available. All images were anonymized and presented in random order. As the memory washout period, the interval between the two readings was at least 2 weeks. All readers were blinded to the patient's clinically relevant information and coronary angiography results. Coronary artery stenosis was assessed in segments with a diameter of ≥ 1.5 mm based on the 18-segment Society of Cardiovascular Computed Tomography (SCCT) model (15). The estimation of the coronary stenosis was assessed visually. The degree of lumen stenosis was divided into the following 7 categories: 1-normal: no plaque and no luminal stenosis; 2-slight stenosis: plaque present, luminal stenosis: <25%; 3-mild stenosis: 25–49%; 4-moderate stenosis: 50–69%; 5-severe stenosis: 70–99%; 6-occlusion; 7- uninterpretable due to the presence of calcification. Patients with a percentage stenosis of <50% were classified as the stenosis (–) group, while those with a percentage stenosis of ≥50% were classified as the stenosis (+) group. Uninterpretable segments were recorded and considered to be stenotic in the accuracy analysis. Image quality was evaluated by using a 4-point scale. Severe calcification was defined as a cross-section calcification artifact arc ≥180° (16). Diagnostic confidence in the assessment of the degree of luminal stenosis was rated using a 5-point Likert scale as follows: 1-very low; 2-low; 3-moderate; 4-high; and 5-very high.

All ICA images were interpreted by two expert interventional cardiologists with more than 10 years of clinical ICA experience who were blinded to all patient characteristics and CCTA findings. Any discrepancy between the observers was settled by consensus. Stenosis equivalent to at least 50% of the diameter was defined as obstructive CAD for ICA and CCTA.

### Statistical Analysis

Statistical analysis was performed by using SPSS software (version 26). Diagnostic confidence was computed for each radiologist and each reading group with different levels of experience. Group differences in diagnostic confidence were compared by using Student's *t*-test. Taking the ICA results as the standard reference, the sensitivity, specificity, positive predictive value (PPV), and negative predictive value (NPV) of conventional and subtraction CCTA were calculated for each radiologist and each reading group. Because of the presence of paired samples, the McNemar test was applied to explore group differences in sensitivity and specificity. The overall diagnostic accuracy was measured with respect to the ICA results by the AUC using the total number of points. The AUCs were compared by using the Hanley-McNeil method (17). All tests were two tailed, and the significance threshold was *p* < 0.05.

## Results

### Baseline Information

**Figure 2:**
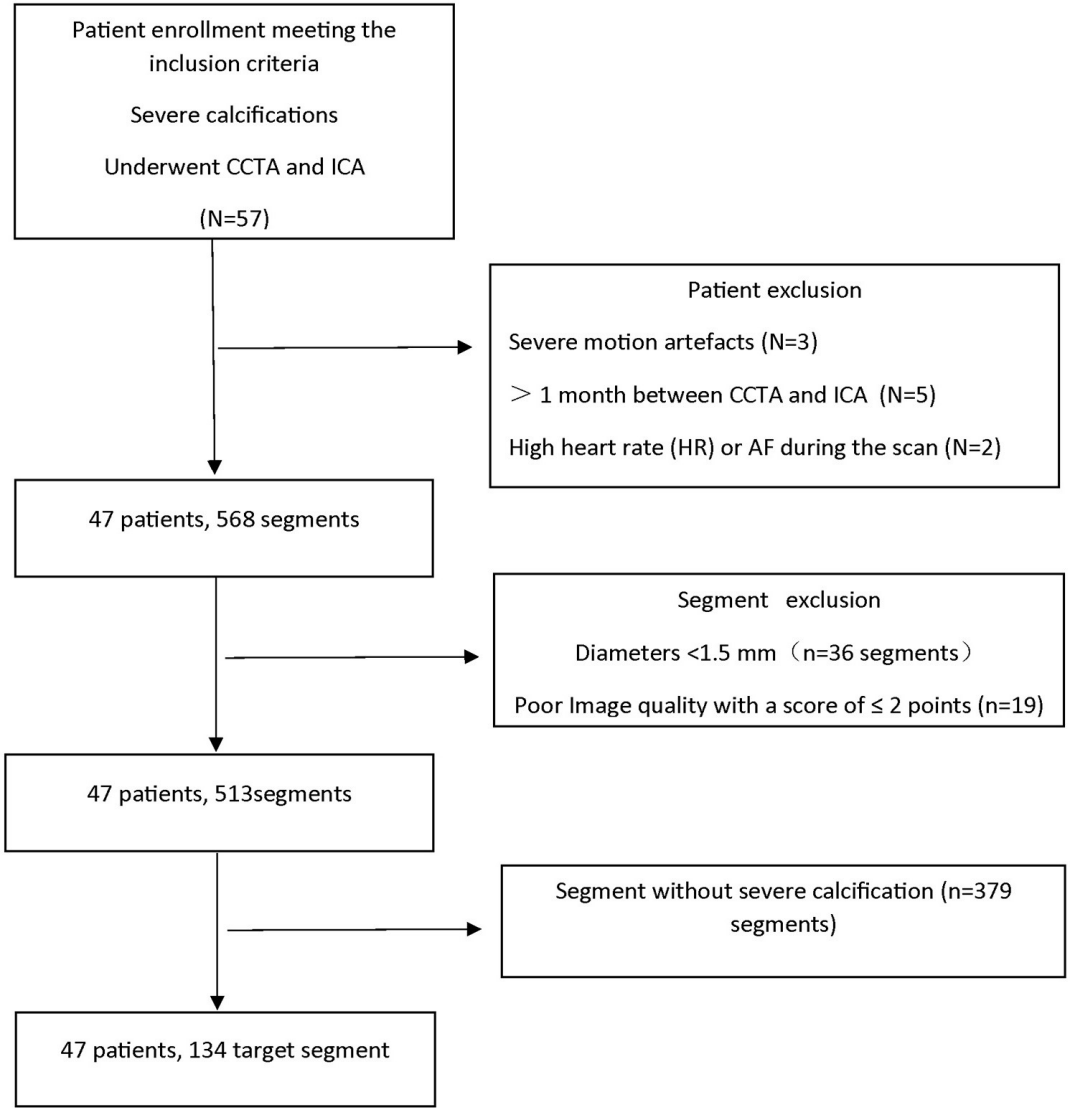
Flowchart of patient enrollment. CCTA, coronary computed tomography angiography; ICA, invasive coronary angiography; AF, atrial fibrillation; *N* represents the number of patients; *n* represents the number of segments.

Forty-seven patients (26 men and 21 women) were enrolled, with a mean age of 67.1 ± 7.3 years. The median Agatston score was 427.1 (IQR: 235.4–886.3). The baseline characteristics of the patients are summarized in Table 1. After exclusion at the patient level, we performed another exclusion at the segment level. Out of a total of 568 segments, 36 segments had diameters <1.5 mm, and 19 segments were excluded because the image quality was <2 points. Of the remaining 513 segments, 379 segments were excluded because there were no target segments. Finally, 134 severely calcified target segments were analyzed, including 49 eccentric calcification and 85 concentric calcification. Among the 134 target segments in this study, 87 segments were well-coregistered, 47 segments had different degrees of misregistration. The enrollment workflow is summarized in Figure 2.

**Table 1:** Baseline patient characteristics (*n* = 47).

**Variables**	**Values**
Men (%)	26 (55.3)
Age (years)	67.1 ± 7.3
Heart rate (bpm)	62.3 ± 6.5
Body mass index (kg/m^2^)	26.9 ± 3.6
Hypertension (*n*, %)	39 (82.3)
Hyperlipidemia (*n*, %)	43 (91.5)
Smoking (*n*, %)	28 (59.6)
Diabetes mellitus (*n*, %)	22 (46.8)
Family history of coronary artery disease (*n*, %)	14 (29.8)
Total effective dose (mSv, median, IQR)	4.5 (3.9–5.4)
Agatston score (median, IQR)	427.1 (235.4–886.3)

### Diagnostic Confidence for Novice, Junior, and Senior Radiologists

**Figure 3:**
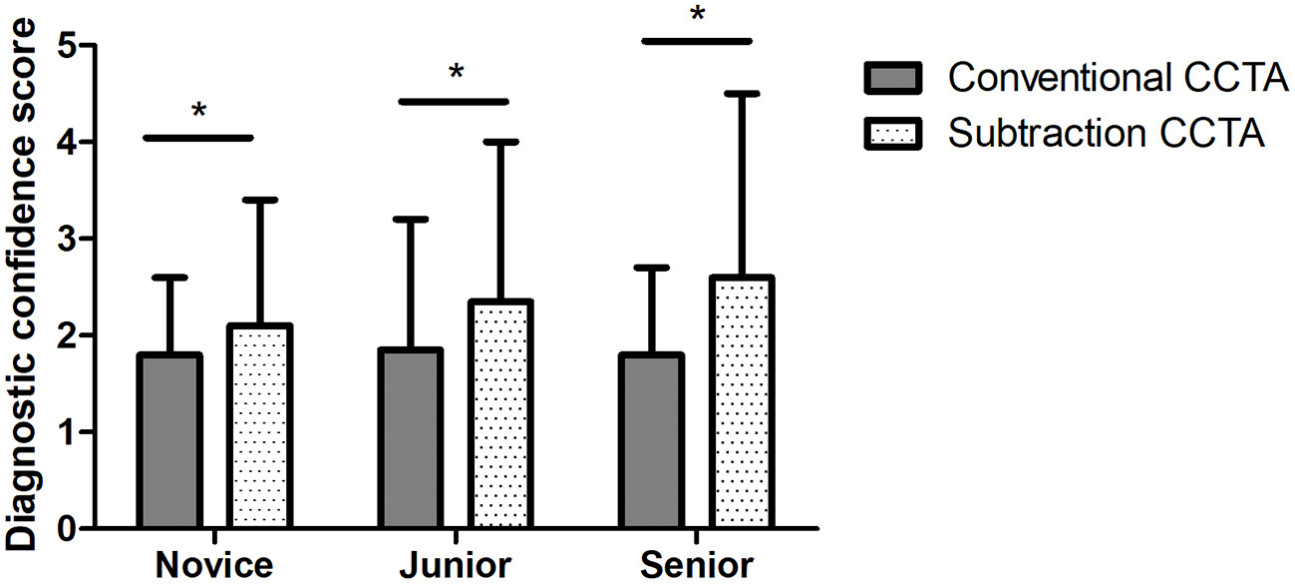
Mean diagnostic confidence with conventional and subtraction coronary computed tomography angiography (CCTA) for novice, junior, and senior radiologists. The chart shows that the mean diagnostic confidence of subtraction CCTA is significantly higher than that of conventional CCTA, with *p* < 0.001 for all three groups. The * symbol indicates the value *p* < 0.01.

For the conventional CCTA data, the mean diagnostic confidence was 2.6 ± 1.0, 3.2 ± 0.5, and 2.7 ± 0.9 for the novice, junior, and senior reading groups, respectively. Comparatively, each reading group showed significantly stronger diagnostic confidence with subtraction CCTA data, with mean values of 3.4 ± 0.8, 4.0 ± 0.7, and 4.5 ± 0.7, respectively (all *p* < 0.001, Figure 3).

The diagnostic confidence of each reader significantly increased after calcifications were subtracted (all *p* < 0.001, Table 2).

**Table 2:** Diagnostic confidence for each radiologist with conventional or subtraction CCTA.

**Radiologist No**.	**Conventional CCTA**		**Subtraction CCTA**		***P*** **-value**
	**Mean**	**SD**	**Mean**	**SD**	
**Novice**	
1	1.9	0.9	3.3	0.9	<0.001
2	3.2	0.5	3.5	0.6	<0.001
3	2.6	1.0	3.5	1.0	<0.001
Mean	2.6	1.0	3.4	0.8	<0.001
**Junior**	
1	3.3	0.5	4.3	0.7	<0.001
2	3.1	0.5	3.7	0.7	<0.001
Mean	3.2	0.5	4.0	0.7	<0.001
**Senior**	
1	2.7	1.0	4.6	0.6	<0.001
2	2.8	0.8	4.3	0.7	<0.001
3	2.6	1.1	4.6	0.6	<0.001
Mean	2.7	0.9	4.5	0.7	<0.001

*CCTA, coronary computed tomography angiography; SD, standard deviation*.

### Comparison Between the Sensitivity, Specificity, PPV, and NPV of Conventional and Subtraction CCTA for Radiologists With Different Levels of Experience

When using conventional CCTA, 9, 4, and 9 segments were rated as uninterpretable by at least one reader in the novice, junior and senior reading groups, respectively. However, when information from subtraction CCTA was included, the number of uninterpretable segments decreased to 4, 2, and 0 for each group.

As shown in Table 3, the mean sensitivity of conventional CCTA for each group ranged from 83.3 to 88.0%. Following calcification subtraction, the mean sensitivity decreased for the novice (*p* < 0.001) and junior radiologists (*p* = 0.017) but remained similar for the senior radiologists (*p* = 0.690). The mean specificity of conventional CCTA for each group ranged from 22.4 to 42.2%, which significantly improved following the inclusion of subtraction CCTA, ranging from 81.3 to 85.7% (all *p* < 0.001). The mean PPVs and NPVs of subtraction CCTA were both higher than those of conventional CCTA.

**Table 3:** Diagnostic performance in detecting obstructive CAD for novice, junior, and senior radiologists with conventional or subtraction CCTA.

**Diagnostic performance**	**Novice (three radiologists)**			**Junior (two radiologists)**			**Senior (three radiologists)**		
	**Con**	**Sub**	***P*** **-value**	**Con**	**Sub**	***P*** **-value**	**Con**	**Sub**	***P*** **-value**
Sensitivity (%)	88.0 (86.0–93.5)	63.9 (54.7–73.1)	<0.001	83.3 (73.6–90.3)	66.7 (55.6–77.8)	0.017	83.3 (75.9–89.8)	86.1 (79.6–92.6)	0.690
Specificity (%)	22.4 (17.7–27.2)	81.3 (76.9–86.1)	<0.001	29.1 (23.0–35.2)	85.7 (80.6–90.3)	<0.001	42.2 (36.7–48.0)	84.7 (80.6–88.8)	<0.001
PPV (%)	29.4 (24.5–34.1)	55.6 (46.0–64.5)	-	30.2 (24.1–36.7)	63.2 (51.3–73.7)	-	34.6 (28.8–40.8)	67.4 (59.4–75.4)	-
NPV (%)	83.5 (74.7–91.1)	86.0 (81.7–89.6)	-	82.6(73.9–91.3)	87.5 (82.8–91.7)	-	87.3 (81.7–92.3)	94.3 (91.3–97.0)	-

*Data in parentheses are 95% confidence intervals*.

*CAD, coronary artery disease; Con, conventional CCTA; Sub, subtraction CCTA; PPV, positive predictive value; NPV, negative predictive value*.

### Comparison Between the AUCs of Conventional and Subtraction CCTA for Radiologists With Different Levels of Experience

**Figure 4:**
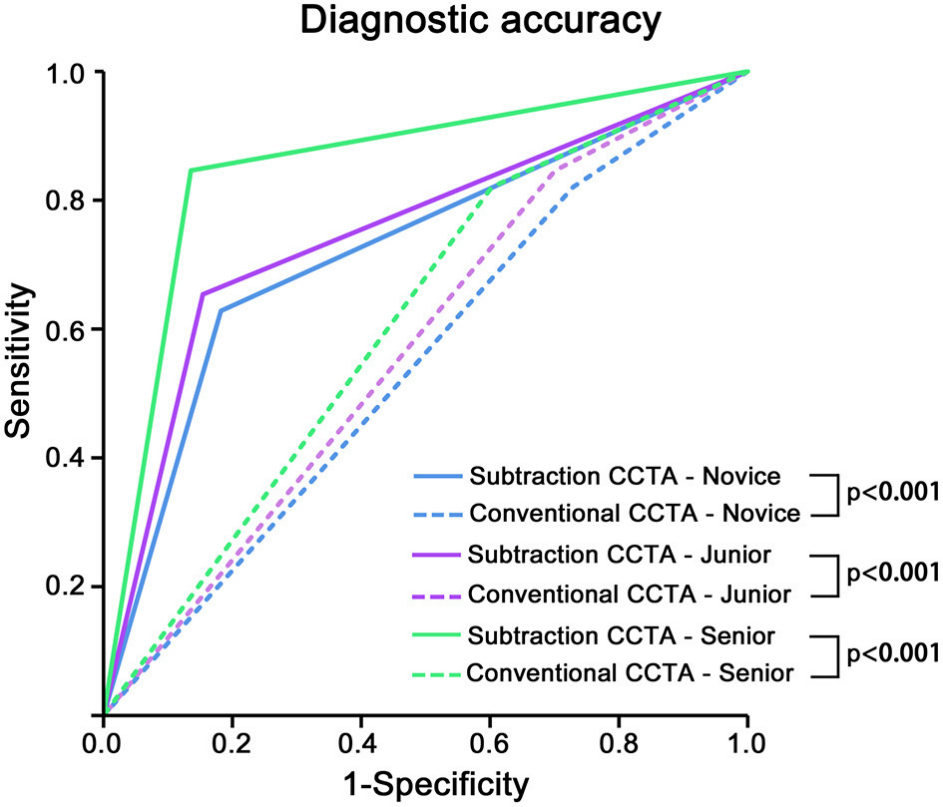
The overall diagnostic accuracy of conventional and subtraction coronary computed tomography angiography (CCTA) for novice, junior, or senior radiologists. The overall diagnostic accuracy was calculated by the area under the curve (AUC). The solid line represents subtraction CCTA, and the dashed line represents conventional CCTA. The colors green, blue and purple represent the novice, junior, and senior radiologists, respectively. The AUC of subtraction CCTA was significantly higher than that of conventional CCTA in each radiologist group stratified by working experience (all *p* < 0.001).

The overall diagnostic accuracy of CCTA was estimated by computing the AUC. The mean AUCs of conventional CCTA for novice, junior and senior radiologists were 0.55 (95% CI: 0.49–0.61), 0.56 (95% CI: 0.49–0.64), and 0.63 (95% CI: 0.57–0.69), respectively. Comparatively, the mean AUCs of subtraction CCTA were significantly increased (all *p* < 0.001), with values of 0.73 (95% CI: 0.67–0.79), 0.76 (95% CI: 0.69–0.83), and 0.85 (95% CI: 0.81–0.90) for the respective groups (Table 4, Figure 4).

**Table 4:** The area under the curve (AUC) for each radiologist with conventional or subtraction CCTA.

**Radiologist No**.	**Conventional CCTA**	**Subtraction CCTA**	***P*** **-value**
**Novices**	
1	0.55 (0.45–0.66)	0.77 (0.68–0.87)	<0.001
2	0.54 (0.43–0.65)	0.69 (0.58–0.80)	0.021
3	0.56 (0.46–0.67)	0.71 (0.61–0.82)	0.001
Mean	0.55 (0.49–0.61)	0.73 (0.67–0.79)	<0.001
**Junior**	
1	0.54 (0.43–0.64)	0.80 (0.71–0.90)	<0.001
2	0.59 (0.49–0.69)	0.72 (0.61–0.83)	0.015
Mean	0.56 (0.49–0.64)	0.76 (0.69–0.83)	<0.001
**Senior**	
1	0.63 (0.53–0.73)	0.87 (0.80–0.95)	<0.001
2	0.61 (0.50–0.71)	0.83 (0.75–0.92)	<0.001
3	0.65 (0.55–0.75)	0.86 (0.78–0.93)	<0.001
Mean	0.63 (0.57–0.69)	0.85 (0.81–0.90)	<0.001

*Data in parentheses are 95% confidence interval*.

At the individual-reader level, significant increases in the AUC were observed for all radiologists (all *p* < 0.05, Table 4).

As shown in Table 5, in the subgroup analysis of eccentric calcification and central calcification, we found that for concentric calcification, subtraction can significantly improve the AUC for novice, junior and senior radiologists, and for eccentric calcification, the AUC for senior radiologists was significantly improved, while the AUC for novice and junior radiologists was not significantly improved.

**Table 5:** Subgroup analysis of diagnostic accuracy of eccentric calcification and concentric calcification by readers with different experience.

**Diagnostic performance**	**Eccentric calcification (*****n*** **= 49)**			**Concentric calcification (*****n*** **= 85)**		
	**Con**	**Sub**	***P*** **-value**	**Con**	**Sub**	***P*** **-value**
**Novice**	
Sensitivity (%)	88.0 (72.2–100.0)	44.4 (22.2–66.7)	0.021	87.8 (81.1–94.4)	67.8 (58.9–77.8)	0.001
Specificity (%)	26.4 (18.6–34.1)	86.8 (80.6–92.2)	<0.001	19.4 (13.3–26.1)	77.0 (70.9–83.6)	<0.001
PPV (%)	14.4 (8.1–20.7)	32.0 (16.0–52.0)	-	37.3 (30.7–43.4)	61.6 (52.5–70.7)	-
NPV (%)	94.4 (86.1–100.0)	91.8 (86.9–96.7)	-	74.4 (62.8–86)	81.4 (75–87.8)	-
AUC	0.58 (0.45–0.71)	0.66 (0.51–0.81)	0.295	0.54 (0.46–0.61)	0.72 (0.66–0.79)	<0.001
**Junior**	
Sensitivity (%)	66.7 (41.7–91.7)	41.7 (16.7–66.7)	0.250	86.7 (78.3–95.0)	71.7 (60.0–81.7)	0.64
Specificity (%)	37.2 (27.9–47.7)	89.5 (82.6–95.3)	<0.001	22.7 (15.5–30.9)	82.7 (75.5–90.0)	<0.001
PPV (%)	12.9 (4.8–22.6)	35.7 (14.3–64.3)	-	38.0 (29.2–46.0)	69.4 (58.1–80.6)	-
NPV (%)	88.9 (77.8-97.2)	91.7 (85.7–96.4)	-	75.8 (60.6–90.9)	84.3 (76.9–90.7)	-
AUC	0.52 (0.35–0.69)	0.66 (0.47–0.84)	0.053	0.55 (0.46–0.64)	0.77 (0.69–0.85)	<0.001
**Senior**	
Sensitivity (%)	66.7 (44.4–88.9)	88.9 (72.2–100.0)	0.219	86.7 (78.9–93.3)	85.6 (78.9–92.2)	1.00
Specificity (%)	57.4 (48.1–65.9)	89.1 (83.7–93.8)	<0.001	30.3 (23.0–37.6)	81.2 (75.2–86.7)	<0.001
PPV (%)	17.9 (9.0–28.4)	53.3 (33.4–70.0)	-	40.4 (33.7–47.7)	71.3 (62.0–79.6)	-
NPV (%)	92.5 (86.3–97.5)	98.3 (95.7–100.0)	-	80.6 (71.0–90.3)	91.2 (86.4–95.2)	-
AUC	0.62 (0.48–0.76)	0.89 (0.80–0.98)	<0.001	0.59 (0.51–0.66)	0.83 (0.78–0.89)	<0.001

*Data in parentheses are 95% confidence interval*.

## Discussion

Since calcified lesions are associated with blooming and beam-hardening artifacts and partial volume effects, the assessment of luminal stenosis is often difficult or impossible (7, 18–20). Some previous studies have shown that the application of subtraction CCTA can reduce the number of uninterpretable segments, improve image quality and improve diagnostic performance (9, 21). However, in those studies, CCTA data were analyzed exclusively by experienced cardiovascular radiologists, and it remains to be ascertained whether high diagnostic performance can also be achieved in readers with limited CTA experience. In this article, we simulate scenarios of the application of subtraction CCTA for readers with different levels of experience. The most significant findings of the present study were that subtraction CCTA reduced the number of uninterpretable segments with severe calcification. Furthermore, subtraction CCTA improved the diagnostic accuracy and confidence of readers with different levels of experience in coronary angiography interpretation at both the individual and group levels.

Research by Amanuma et al. (10) shows that when the information provided by subtraction CCTA was added, the number of non-assessable segments was reduced from 74 to 18. Consistent with previous findings, we demonstrated that the numbers decreased from 9, 4, and 9 to 4, 2, and 0 for each group.

De Santis et al. (22) scored diagnostic confidence using 5-point Likert scales. Their research shows that the calcium subtraction algorithm improved diagnostic confidence and that the mean score increased from 3.1 to 4.0. From our research, we can also see that calcification had an impact on physicians' diagnostic confidence. Regardless of whether they were experienced, the confidence in the CCTA diagnosis of severely calcified segments was not high. After calcium subtraction, the diagnostic confidence significantly improved at both the individual and group levels.

The quality of CCTA interpretation increases with the level of experience (23). It is unclear whether there are risks or benefits to expanding the clinical use of subtraction CCTA among inexperienced readers. According to our research results, with subtraction CCTA information, the mean specificity of senior radiologists was significantly improved, and the sensitivity was not decreased, which was similar to most previous research results. The study by Amanuma et al. (10) showed that the specificity was increased from 54.5 to 78.5%, but the sensitivity was not changed. A multicenter study (9) showed that the subtraction procedure lowered the false-positive rate in target segments without misregistration from 72 to 33% (false positives equal to 1-specificity) at the expense of 7% false negatives in subtraction CCTA. Different from these findings, it was also observed in our study that the novice and junior radiologists showed significant increases while the sensitivity was decreased. We deem the reason for this phenomenon is that all segments interpreted in our study were severely calcified segments; however, with the conventional CCTA images, the novice and junior radiologists overestimated the degree of stenosis, and the incidence of coronary artery stenosis in severely calcified segments was relatively high, resulting in exaggerated sensitivity. The previous literature shows that the sensitivity of inexperienced radiologists is significantly lower than that of experienced radiologists in the interpretation of routine CCTA (23), while the sensitivity of novice and junior radiologists in our results was no less than that of senior radiologists because the target segments in our study were severely calcified. Severe calcification results in increased false positives (low specificity) and decreased false negatives (high sensitivity). Therefore, the sensitivity of novice and junior radiologists in our study was exaggerated when only the conventional CCTA images were read. When adding subtraction information, the calcification was removed, and the lumen was exposed to all readers, whose sensitivity and specificity reflected their true ability to interpret CCTA.

Significant increases in AUC values were observed in novice, junior and senior radiologists, suggesting that calcification subtraction was beneficial for all radiologists with different experience levels. Senior readers performed better than novices in evaluating lumen stenosis on subtraction CCTA images, especially in eccentrically calcified segments. For eccentric calcification, part of the lumen was usually obscured by calcification artifacts, which required multiangle observation, which depended on the experience of the reader.

Our current study is unique in that we analyzed the influence of subtraction CCTA on the performance of readers at various experience levels with CCTA interpretation. Following the inclusion of subtraction CCTA images, the specificity and PPV were significantly improved in the novice, junior and senior radiologist groups, and the AUC and diagnostic confidence were significantly improved at the individual and group levels. This means that subtraction CCTA can benefit readers of all levels. Calcification subtraction not only further improves the diagnostic accuracy of senior radiologists, but also novice and junior radiologists to achieve a higher level of accuracy in the diagnosis of severe calcification lesions. Therefore, we recommend the subtraction CCTA to all levels of readers.

In subtraction CCTA, there are two methods for obtaining the non-contrast CT image data. One is a single breath-hold method and the other is a two breath-hold method. Both methods have their own advantages and disadvantages (12, 13). The single breath hold method would require a single breath-hold of 20–40 s. Considering that long-term breath-holding may be difficult for elderly patients with severe coronary calcification, we used the two breath-hold method described by Yoshioka et al. (8). Among the 134 target segments in this study, 87 segments were well-coregistered, and 47 segments had different degrees of misregistration. The misregistration artifacts in this study was 35% (47/134), which was significantly lower than that of the multicenter study by Fuchs et al. (35 vs. 50%) (9), and close to that of the study by Yi et al. (35 vs. 32.5%) (24), both of which used the two breath-hold method. When the readers interpreted CCTA for the second time, the subtraction CCTA image was used as a supplement to the conventional CCTA image. Therefore, we did not exclude misaligned segments. This may be a limitation of this study, but this is more concordant with the clinical routine. Whether it is the one-breath-hold method or the two-breath-hold method, misregistration is inevitable. We should take measures to minimize the misregistration, but despite the presence of misregistered segments, readers with different experience can benefit from subtraction CCTA, which shows that subtraction CCTA is useful. As dual-energy CT without the need for additional image acquisition, the problems regarding misregistration artifacts could be solved (22). The second limitation is that the target segment in this paper is the severely calcified segment defined by visual assessment. The Agatston scores of some patients were not high. Although the Agatston score is more objective than visual methods, the segment calcium severity is not linearly associated with the patient's Agatston score (25). The third limitation is that the sample size of interpreters was not large enough. In future research, we will enroll more samples and conduct a multicenter study.

## Conclusion

Subtraction CCTA could improve the diagnostic accuracy and diagnostic confidence of readers with different levels of experience in coronary CT angiography interpretation. Not only the senior readers with rich experience, but also readers with less experience.

## Data Availability Statement

The original contributions presented in the study are included in the article/supplementary materials, further inquiries can be directed to the corresponding authors.

## Ethics Statement

The studies involving human participants were reviewed and approved by Ethics Committee of Beijing Friendship Hospital, Capital Medical University. The patients/participants provided their written informed consent to participate in this study.

## Author Contributions

YH, ZY, ZW, and YX were contributed to the conception, design, and analyzed the data. FL and QH were contributed to the experiments and wrote the draft manuscript. LX was contributed to methodology statistics and edited the manuscript. YZ and YS were contributed to study implement. All authors approved the final manuscript submitted.

## Funding

This work was supported by the National Key Research and Development Program of China (grant 2019YFE0107800), National Natural Science Foundation of China (grant 81971569), and Beijing Municipal Science and Technology Commission (grant Z201100005620009).

## Conflict of Interest

YX was employed by Canon Medical Systems (China) Co. Ltd. The remaining authors declare that the research was conducted in the absence of any commercial or financial relationships that could be construed as a potential conflict of interest.

## Publisher's Note

All claims expressed in this article are solely those of the authors and do not necessarily represent those of their affiliated organizations, or those of the publisher, the editors and the reviewers. Any product that may be evaluated in this article, or claim that may be made by its manufacturer, is not guaranteed or endorsed by the publisher.

## Abbreviations

ICA: invasive coronary angiography
CAD: coronary artery disease
CCTA: coronary computed tomography angiography
CT: computed tomography
AUC: area under the curve
PPV: positive predictive value
NPV: negative predictive value.

## References

[1] Serruys PW Hara H Garg S Kawashima H Nørgaard BL Dweck MR. Coronary computed tomographic angiography for complete assessment of coronary artery disease: JACC state-of-the-art review. J Am Coll Cardiol. (2021) 78:713–36. 10.1016/j.jacc.2021.06.019 34384554

[2] Giusca S Schütz M Kronbach F Wolf D Nunninger P Korosoglou G. Coronary computer tomography angiography in 2021-acquisition protocols, tips and tricks and heading beyond the possible. Diagnostics. (2021) 2021:1072. 10.3390/diagnostics11061072 34200866 PMC8230532

[3] Pugliese F Hunink MG Gruszczynska K Alberghina F Malagó R van Pelt N. Learning curve for coronary CT angiography: what constitutes sufficient training? Radiology. (2009) 251:359–68. 10.1148/radiol.2512080384 19401570

[4] Schroeder S Achenbach S Bengel F Burgstahler C Cademartiri F de Feyter P. Cardiac computed tomography: indications, applications, limitations, and training requirements: report of a Writing Group deployed by the Working Group Nuclear Cardiology and Cardiac CT of the European Society of Cardiology and the European Council of Nuclear Cardiology. Eur Heart J. (2008) 29:531–56. 10.1093/eurheartj/ehm544 18084017

[5] Maroules CD Hamilton-Craig C Branch K Lee J Cury RC Maurovich-Horvat P. Coronary artery disease reporting and data system (CAD-RADS(TM)): inter-observer agreement for assessment categories and modifiers. J Cardiovasc Comput Tomogr. (2018) 12:125–30. 10.1016/j.jcct.2017.11.014 29217341

[6] Andrew M John H. The challenge of coronary calcium on coronary computed tomographic angiography (CCTA) scans: effect on interpretation and possible solutions. Int J Cardiovasc Imaging. (2015) 31:145–57. 10.1007/s10554-015-0773-0 26408105

[7] Vavere AL Arbab-Zadeh A Rochitte CE Dewey M Niinuma H Gottlieb I. Coronary artery stenoses: accuracy of 64-detector row CT angiography in segments with mild, moderate, or severe calcification–a subanalysis of the CORE-64 trial. Radiology. (2011) 261:100–8. 10.1148/radiol.11110537 21828192 PMC3176425

[8] Yoshioka K Tanaka R Muranaka K. Subtraction coronary CT angiography for calcified lesions. Cardiol Clin. (2012) 30:93–102. 10.1016/j.ccl.2011.10.004 22304952

[9] Fuchs A Kühl JT Chen MY Viladés Medel D Alomar X Shanbhag SM. Subtraction CT angiography improves evaluation of significant coronary artery disease in patients with severe calcifications or stents-the C-Sub 320 multicenter trial. Eur Radiol. (2018) 28:4077–85. 10.1007/s00330-018-5418-y 29696430 PMC6737932

[10] Amanuma M Kondo T Sano T Sekine T Takayanagi T Matsutani H. Subtraction coronary computed tomography in patients with severe calcification. Int J Cardiovasc Imaging. (2015) 31:1635–42. 10.1007/s10554-015-0746-3 26288954

[11] Fuchs A Kühl JT Chen MY Helqvist S Razeto M Arakita K. Feasibility of coronary calcium and stent image subtraction using 320-detector row CT angiography. J Cardiovasc Comput Tomogr. (2015) 9:393–8. 10.1016/j.jcct.2015.03.016 26091841 PMC6290475

[12] Viladés Medel D Leta R Alomar Serralach X Carreras Costa F Pons-Lladó G. Reliability of a new method for coronary artery calcium or metal subtraction by 320-row cardiac CT. Eur Radiol. (2016) 26:3208–14. 10.1007/s00330-015-4130-4 26662029

[13] Yoshioka K Tanaka R Muranaka K Sasaki T Ueda T Chiba T. Subtraction coronary CT angiography using second-generation 320-detector row CT. Int J Cardiovasc Imaging. (2015) 31:51–8. 10.1007/s10554-015-0630-1 25721727

[14] Tanaka R Yoshioka K Muranaka K Chiba T Ueda T Sasaki T. Improved evaluation of calcified segments on coronary CT angiography: a feasibility study of coronary calcium subtraction. Int J Cardiovasc Imaging. (2013) 29:75–81. 10.1007/s10554-013-0316-5 24158235

[15] Wu F-Z Wu M-T. 2014 SCCT guidelines for the interpretation and reporting of coronary CT angiography: a report of the Society of Cardiovascular Computed Tomography Guidelines Committee. J Cardiovasc Comput Tomogr. (2015) 9:e3. 10.1016/j.jcct.2015.01.003 25708015

[16] Xu L Li F Wu K Xu L Li F Wu K. Subtraction improves the accuracy of coronary CT angiography for detecting obstructive disease in severely calcified segments. Eur Radiol. (2021) 31:6211–9. 10.1007/s00330-021-08092-5 34142220

[17] Hanley JA McNeil BJ. A method of comparing the areas under receiver operating characteristic curves derived from the same cases. Radiology. (1983) 148:839–43. 10.1148/radiology.148.3.6878708 6878708

[18] Budoff MJ Dowe D Jollis JG Gitter M Sutherland J Halamert E. Diagnostic performance of 64-multidetector row coronary computed tomographic angiography for evaluation of coronary artery stenosis in individuals without known coronary artery disease: results from the prospective multicenter ACCURACY (Assessment by Coronary Computed Tomographic Angiography of Individuals Undergoing Invasive Coronary Angiography) trial. J Am Coll Cardiol. (2008) 52:1724–32. 10.1016/j.jacc.2008.07.031 19007693

[19] Abdulla J Pedersen KS Budoff M Kofoed KF. Influence of coronary calcification on the diagnostic accuracy of 64-slice computed tomography coronary angiography: a systematic review and meta-analysis. Int J Cardiovasc Imaging. (2012) 28:943–53. 10.1007/s10554-011-9902-6 21667273

[20] Brodoefel H Burgstahler C Tsiflikas I Reimann A Schroeder S Claussen CD. Dual-source CT: effect of heart rate, heart rate variability, and calcification on image quality and diagnostic accuracy. Radiology. (2008) 247:346–55. 10.1148/radiol.2472070906 18372455

[21] Guo W Tripathi P Yang S Qian J Rai B Zeng M. Modified subtraction coronary CT angiography with a two-breathhold technique: image quality and diagnostic accuracy in patients with coronary calcifications. Korean J Radiol. (2019) 20:1146–55. 10.3348/kjr.2018.0845 31270978 PMC6609439

[22] De Santis D Jin KN Schoepf UJ Grant KL De Cecco CN Nance JW Jr. Heavily calcified coronary arteries: advanced calcium subtraction improves luminal visualization and diagnostic confidence in dual-energy coronary computed tomography angiography. Invest Radiol. (2018) 53:103–9. 10.1097/RLI.0000000000000416 29016370

[23] Kerl JM Schoepf UJ Bauer RW Tekin T Costello P Vogl TJ. 64-slice multidetector-row computed tomography in the diagnosis of coronary artery disease: interobserver agreement among radiologists with varied levels of experience on a per-patient and per-segment basis. J Thorac Imaging. (2012) 27:29–35. 10.1097/RTI.0b013e3181f82805 21102356

[24] Yi Y Xu C Xu M Yan J Li YY Wang J. Diagnostic improvements of deep learning-based image reconstruction for assessing calcification-related obstructive coronary artery disease. Front Cardiovasc Med. (2021) 8:758793. 10.3389/fcvm.2021.758793 34805313 PMC8595262

[25] Hong C Pilgram TK Zhu F Bae KT. Is coronary artery calcium mass related to Agatston score? Acad Radiol. (2004) 11:286–92. 10.1016/S1076-6332(03)00714-1 15035519

